# 4-(9-Anthr­yl)-1-(2,4-dimethoxy­phen­yl)spiro­[azetidine-3,9′-xanthen]-2-one

**DOI:** 10.1107/S1600536809022739

**Published:** 2009-06-20

**Authors:** Zeliha Baktır, Mehmet Akkurt, Aliasghar Jarrahpour, Edris Ebrahimi, Orhan Büyükgüngör

**Affiliations:** a Department of Physics, Faculty of Arts and Sciences, Erciyes University, 38039 Kayseri, Turkey; b Department of Chemistry, College of Sciences, Shiraz University, 71454 Shiraz, Iran; c Department of Physics, Faculty of Arts and Sciences, Ondokuz Mayıs University, 55139 Samsun, Turkey

## Abstract

The title compound, C_37_H_27_NO_4_, crystallizes with two mol­ecules in the asymmetric unit. The β-lactam ring of each mol­ecule is very nearly planar, with maximum deviations of 0.001 (2) and 0.017 (2) Å in the two mol­ecules. The crystal structure is stabilized by inter­molecular C—H⋯O and C—H⋯N contacts, as well as by weak C—H⋯π inter­actions.

## Related literature

For the biological properties of spiro-β-lactams, see: Kobayashi *et al.* (1991 ▶); Sheehan *et al.* (1978 ▶); Skiles & McNeil (1990 ▶); Waldmann (1995 ▶). For polycyclic aromatic β-lactams with anti-cancer activity, see: Banik *et al.* (2003 ▶, 2004 ▶); Becker & Banik (1998 ▶). For several syntheses of spiro-β-lactams, see: Jarrahpoor & Khalili (2007 ▶). For the structural characterizations of some β-lactam compounds, see: Akkurt *et al.* (2008*a* ▶,*b* ▶); Yalçın *et al.* (2009 ▶). For ring puckering analysis, see: Cremer & Pople (1975 ▶).
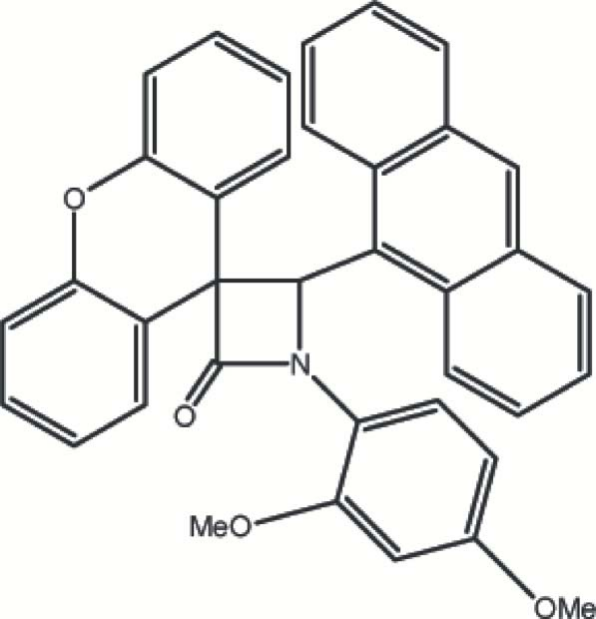


## Experimental

### 

#### Crystal data

C_37_H_27_NO_4_
*M* *_r_* = 549.60
Triclinic, 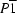

*a* = 12.3164 (6) Å
*b* = 13.1277 (7) Å
*c* = 18.4838 (11) Å
α = 92.434 (5)°
β = 109.236 (4)°
γ = 91.357 (4)°
*V* = 2816.9 (3) Å^3^
*Z* = 4
Mo *K*α radiation
μ = 0.08 mm^−1^
*T* = 295 K
0.35 × 0.32 × 0.28 mm

#### Data collection

Stoe IPDS 2 diffractometer
Absorption correction: integration (*X-RED32*; Stoe & Cie, 2002 ▶) *T*_min_ = 0.971, *T*_max_ = 0.977
30264 measured reflections
11851 independent reflections
6076 reflections with *I* > 2σ(*I*)
*R*_int_ = 0.100

#### Refinement

*R*[*F* ^2^ > 2σ(*F* ^2^)] = 0.057
*wR*(*F* ^2^) = 0.131
*S* = 0.88
11851 reflections
762 parameters
2 restraints
H-atom parameters constrained
Δρ_max_ = 0.18 e Å^−3^
Δρ_min_ = −0.23 e Å^−3^

### 

Data collection: *X-AREA* (Stoe & Cie, 2002 ▶); cell refinement: *X-AREA*; data reduction: *X-RED32* (Stoe & Cie, 2002 ▶); program(s) used to solve structure: *SHELXS97* (Sheldrick, 2008 ▶); program(s) used to refine structure: *SHELXL97* (Sheldrick, 2008 ▶); molecular graphics: *ORTEP-3* (Farrugia, 1997 ▶); software used to prepare material for publication: *WinGX* (Farrugia, 1999 ▶).

## Supplementary Material

Crystal structure: contains datablocks global, I. DOI: 10.1107/S1600536809022739/tk2470sup1.cif

Structure factors: contains datablocks I. DOI: 10.1107/S1600536809022739/tk2470Isup2.hkl

Additional supplementary materials:  crystallographic information; 3D view; checkCIF report

## Figures and Tables

**Table 1:** Hydrogen-bond geometry (Å, °)

*D*—H⋯*A*	*D*—H	H⋯*A*	*D*⋯*A*	*D*—H⋯*A*
C2—H2⋯N1	0.93	2.33	2.964 (3)	125
C2′—H2′⋯N1′	0.93	2.31	2.957 (3)	126
C3—H3⋯O4′^i^	0.93	2.59	3.484 (4)	163
C9—H9⋯O1^ii^	0.93	2.56	3.458 (4)	164
C35—H35⋯O2	0.93	2.58	3.132 (3)	119
C35—H35⋯O2^iii^	0.93	2.52	3.212 (3)	131
C35′—H35′⋯O2′	0.93	2.52	3.106 (3)	121
C2—H2⋯*Cg*1	0.93	2.59	3.188 (3)	122
C2′—H2′⋯*Cg*15	0.93	2.62	3.196 (3)	121

Symmetry codes: (i) 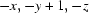; (ii) 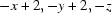; (iii) 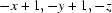. *Cg*1 and *Cg*15 are the centroids of the N1/C15/C16/C29 and N1′/C15′/C16′/C29′ rings, respectively.

**Fig. 1.**
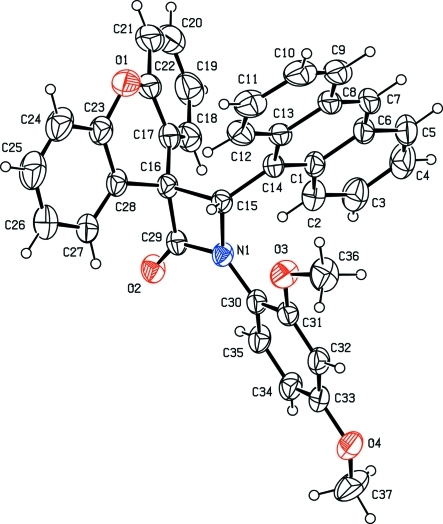
The molecular structure of molecule IA of (I), with the atom-numbering scheme and 30% probability displacement ellipsoids.

**Fig. 2.**
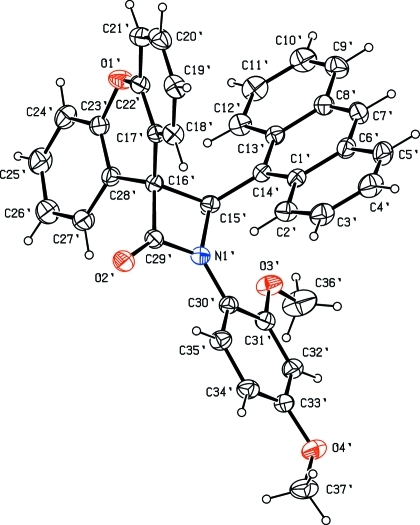
The molecular structure of molecule IB of (I), with the atom-numbering scheme and 30% probability displacement ellipsoids.

## supplementary crystallographic information

### Comment

Spiro compounds represent an important class of naturally occurring compounds characterized by pronounced biological properties (Kobayashi *et al.*, 1991; Waldmann, 1995). Spiro-β-lactams are also interesting due to their anti-viral (Skiles & McNeil, 1990) and anti-bacterial properties (Sheehan *et al.*, 1978). Several syntheses of spiro-β-lactams are available in the literature (Jarrahpoor & Khalili, 2007). Some polycyclic aromatic β-lactams with anti-cancer effects have also been reported (Banik *et al.*, 2003 & 2004; Becker & Banik, 1998). As an extension of our work (Yalçın *et al.*, 2009; Akkurt *et al.*, 2008*a*,*b*) on structural characterization of the β-lactam compounds, the title compound, (I), is reported herein.

The asymmetric unit of (I) contains two enantiomorphic molecules IA and IB (Fig. 1 for molecule IA and Fig. 2 for molecule IB) and there is one chiral centre in each molecule, i.e. at C15, which have S and R configurations. In (I), the bond lengths and angles are comparable with the values found in related structures (Yalçın *et al.*, 2009; Akkurt *et al.*, 2008*a*,*b*). The β-lactam units of molecules IA and IB are very nearly planar, with maximum deviations of 0.001 (2) Å and -0.017 (2) Å, respectively, from the respective least-squares plane. The dihedral angles between the planes in molecules IA and IB are given in Table 2, and those between the similar planes of the molecules IA and IB are given in Table 3.

In the xanthene ring systems, the central ring, [O1/C16/C17/C22/C23/C28 for molecule IA, and O1'/C16'/C17'/C22'/C23'/C28' for molecule IB] is not planar, with puckering parameters: Q_T_= 0.417 (3) Å, θ = 98.2 (4)° and φ = 4.8 (4)° for molecule IA and Q_T_= 0.357 (2) Å, θ = 81.1 (3)° and φ = 185.9 (4)° for molecule IB (Cremer & Pople, 1975).

The anthracene ring systems, attached at C15 for molecule IA and C15' for molecule IB, are almost planar, with significant deviations of -0.068 (4) Å for C3, 0.050 (3) Å for C6, and -0.052 (3) Å for C10 in molecule IA, and with significant deviations of -0.040 (3) Å for C6', 0.043 (3) Å for C11', and -0.075 (2) Å for C14' in molecule IB.

The molecular conformation is stabilized by intramolecular C—H···O and C—H···N contacts. Molecules are linked to each other by intermolecular C—H···O and C—H···N contacts and by weak C—H···π interactions (Table 1).

### Experimental

A mixture of the Schiff base (*E*)—*N*-(anthracen-9-ylmethylene)-2,4-dimethoxybenzamine (0.30 g, 0.87 mmol) and triethylamine (0.44 g, 4.39 mmol), 9*H-*xanthen-9-carboxylic acid (0.30 g,1.31 mmol) and tosyl chloride (0.25 g, 1.31 mmol) in CH_2_Cl_2_ (15 ml) was stirred at room temperature for 24 h. The solution was washed with HCl (1 N, 20 ml), a saturated sodium bicarbonate solution (20 ml), and then brine (20 ml). It was then dried over Na_2_SO_4_. The solvent was evaporated to give the crude product as a light-green crystalline material which was then purified by recrystallization from ethylacetate [yield 69%, m.p. 453–455 K]. IR (KBr, cm^-1^): 1739 (CO β-lactam). ^1^H-NMR δ (p.p.m.): 2.91, 3.58 (s, 6H, 2OMe) 6.16 (s, 1H, 4), 6.17–8.18 (m, ArH, 20H). ^13^C-NMR δ (p.p.m.): 55.4, 55.5(2OMe), 65.9 (C-3), 78.1 (C-4), 100.2–158.2 (aromatic carbon), 167.7 (CO β-lactam). Analysis calculated for C_37_H_27_NO_4_: C 80.86, H 4.95, N 2.55%. Found: C 80.03, H 4.98, N 2.83%

### Refinement

The H atoms were positioned geometrically and refined in a riding model approximation with C—H = 0.93 to 0.98 Å, and with *U*_iso_(H) = 1.2 or 1.5*U*_eq_(C).

### Crystal data

**Table:**

C_37_H_27_NO_4_	*Z* = 4
*M**_r_* = 549.60	*F*(000) = 1152
Triclinic, *P*1	*D*_x_ = 1.296 Mg m^−^^3^
Hall symbol: -P 1	Mo *K*α radiation, λ = 0.71073 Å
*a* = 12.3164 (6) Å	Cell parameters from 23132 reflections
*b* = 13.1277 (7) Å	θ = 1.2–27.3°
*c* = 18.4838 (11) Å	µ = 0.08 mm^−^^1^
α = 92.434 (5)°	*T* = 295 K
β = 109.236 (4)°	Block, light-green
γ = 91.357 (4)°	0.35 × 0.32 × 0.28 mm
*V* = 2816.9 (3) Å^3^	

### Data collection

**Table:**

Stoe IPDS 2 diffractometer	11851 independent reflections
Radiation source: sealed X-ray tube, 12 x 0.4 mm long-fine focus	6076 reflections with *I* > 2σ(*I*)
plane graphite	*R*_int_ = 0.100
Detector resolution: 6.67 pixels mm^-1^	θ_max_ = 26.8°, θ_min_ = 1.8°
ω scans	*h* = −15→15
Absorption correction: integration (*X-RED32*; Stoe & Cie, 2002)	*k* = −16→16
*T*_min_ = 0.971, *T*_max_ = 0.977	*l* = −23→23
30264 measured reflections	

### Refinement

**Table:**

Refinement on *F*^2^	Secondary atom site location: difference Fourier map
Least-squares matrix: full	Hydrogen site location: inferred from neighbouring sites
*R*[*F*^2^ > 2σ(*F*^2^)] = 0.057	H-atom parameters constrained
*wR*(*F*^2^) = 0.131	*w* = 1/[σ^2^(*F*_o_^2^) + (0.0476*P*)^2^] where *P* = (*F*_o_^2^ + 2*F*_c_^2^)/3
*S* = 0.88	(Δ/σ)_max_ = 0.001
11851 reflections	Δρ_max_ = 0.18 e Å^−^^3^
762 parameters	Δρ_min_ = −0.23 e Å^−^^3^
2 restraints	Extinction correction: *SHELXL97* (Sheldrick, 2008), Fc^*^=kFc[1+0.001xFc^2^λ^3^/sin(2θ)]^-1/4^
Primary atom site location: structure-invariant direct methods	Extinction coefficient: 0.0068 (7)

### Special details

**Table:**

Geometry. Bond distances, angles *etc*. have been calculated using the rounded fractional coordinates. All su's are estimated from the variances of the (full) variance-covariance matrix. The cell e.s.d.'s are taken into account in the estimation of distances, angles and torsion angles
Refinement. Refinement on *F*^2^ for ALL reflections except those flagged by the user for potential systematic errors. Weighted *R*-factors *wR* and all goodnesses of fit *S* are based on *F*^2^, conventional *R*-factors *R* are based on *F*, with *F* set to zero for negative *F*^2^. The observed criterion of *F*^2^ > σ(*F*^2^) is used only for calculating -*R*-factor-obs *etc*. and is not relevant to the choice of reflections for refinement. *R*-factors based on *F*^2^ are statistically about twice as large as those based on *F*, and *R*-factors based on ALL data will be even larger.

### Fractional atomic coordinates and isotropic or equivalent isotropic displacement parameters (Å^2^)

**Table:**

	*x*	*y*	*z*	*U*_iso_*/*U*_eq_	
O1	0.8942 (2)	0.88820 (16)	0.18847 (11)	0.0895 (9)	
O2	0.63763 (13)	0.56579 (13)	0.09096 (9)	0.0674 (6)	
O3	0.90833 (14)	0.56385 (14)	−0.05719 (9)	0.0725 (7)	
O4	0.69478 (19)	0.27229 (16)	−0.19830 (10)	0.0908 (8)	
N1	0.75037 (15)	0.59802 (14)	0.01293 (9)	0.0524 (6)	
C1	0.72308 (18)	0.78066 (18)	−0.09183 (11)	0.0592 (9)	
C2	0.6123 (2)	0.7298 (2)	−0.10711 (14)	0.0720 (10)	
C3	0.5219 (3)	0.7432 (3)	−0.17004 (16)	0.0879 (13)	
C4	0.5295 (3)	0.8129 (3)	−0.22451 (17)	0.1029 (15)	
C5	0.6301 (4)	0.8638 (3)	−0.21355 (16)	0.0961 (13)	
C6	0.7287 (3)	0.8506 (2)	−0.14902 (13)	0.0708 (10)	
C7	0.8339 (3)	0.9000 (2)	−0.14035 (15)	0.0786 (13)	
C8	0.9320 (3)	0.88555 (19)	−0.07957 (15)	0.0690 (10)	
C9	1.0389 (3)	0.9364 (2)	−0.07369 (19)	0.0840 (13)	
C10	1.1332 (3)	0.9231 (2)	−0.0159 (2)	0.0913 (15)	
C11	1.1318 (3)	0.8585 (2)	0.04329 (18)	0.0878 (12)	
C12	1.0308 (2)	0.8090 (2)	0.04008 (16)	0.0707 (10)	
C13	0.9280 (2)	0.81834 (17)	−0.02127 (13)	0.0570 (8)	
C14	0.82102 (17)	0.76592 (17)	−0.02899 (11)	0.0541 (8)	
C15	0.82820 (19)	0.69059 (16)	0.03212 (11)	0.0501 (7)	
C16	0.78066 (19)	0.71268 (18)	0.10240 (12)	0.0545 (8)	
C17	0.7174 (2)	0.8073 (2)	0.10394 (13)	0.0634 (10)	
C18	0.5995 (3)	0.8159 (2)	0.06731 (16)	0.0796 (11)	
C19	0.5477 (3)	0.9063 (3)	0.0717 (2)	0.1015 (16)	
C20	0.6127 (5)	0.9907 (3)	0.1132 (2)	0.1125 (18)	
C21	0.7290 (4)	0.9829 (3)	0.1509 (2)	0.1018 (16)	
C22	0.7789 (3)	0.8925 (2)	0.14681 (16)	0.0741 (11)	
C23	0.9323 (2)	0.7946 (2)	0.21506 (15)	0.0747 (11)	
C24	1.0304 (3)	0.7936 (3)	0.27988 (18)	0.1009 (15)	
C25	1.0689 (3)	0.7023 (4)	0.30934 (18)	0.1086 (18)	
C26	1.0104 (3)	0.6127 (3)	0.27628 (16)	0.0884 (13)	
C27	0.9147 (2)	0.6136 (2)	0.21140 (13)	0.0677 (10)	
C28	0.8755 (2)	0.7047 (2)	0.17832 (12)	0.0599 (8)	
C29	0.70803 (19)	0.61445 (18)	0.07177 (12)	0.0532 (8)	
C30	0.73190 (18)	0.51789 (17)	−0.04314 (11)	0.0524 (7)	
C31	0.81466 (19)	0.49726 (18)	−0.07871 (12)	0.0548 (8)	
C32	0.7981 (2)	0.4166 (2)	−0.13046 (13)	0.0648 (9)	
C33	0.7004 (2)	0.3525 (2)	−0.14772 (12)	0.0682 (9)	
C34	0.6163 (2)	0.3748 (2)	−0.11564 (13)	0.0689 (9)	
C35	0.6335 (2)	0.4558 (2)	−0.06368 (12)	0.0637 (9)	
C36	0.9813 (2)	0.5661 (3)	−0.10290 (16)	0.0895 (13)	
C37	0.5967 (3)	0.2041 (3)	−0.2176 (2)	0.1199 (16)	
O1'	0.44211 (14)	0.09851 (14)	0.62126 (8)	0.0719 (6)	
O2'	0.18500 (15)	0.04284 (14)	0.34188 (10)	0.0729 (6)	
O3'	−0.01023 (14)	0.31985 (15)	0.43808 (9)	0.0769 (7)	
O4'	−0.24931 (16)	0.34781 (16)	0.17933 (11)	0.0885 (8)	
N1'	0.13169 (15)	0.19227 (14)	0.39409 (10)	0.0520 (6)	
C1'	0.28673 (18)	0.39075 (16)	0.43460 (11)	0.0508 (7)	
C2'	0.2675 (2)	0.3577 (2)	0.35600 (12)	0.0635 (9)	
C3'	0.2940 (2)	0.4195 (2)	0.30641 (14)	0.0765 (10)	
C4'	0.3409 (2)	0.5182 (3)	0.32963 (18)	0.0871 (12)	
C5'	0.3623 (2)	0.5540 (2)	0.40215 (17)	0.0777 (11)	
C6'	0.3372 (2)	0.49171 (18)	0.45703 (14)	0.0600 (8)	
C7'	0.3643 (2)	0.52860 (19)	0.53249 (15)	0.0689 (10)	
C8'	0.3451 (2)	0.47047 (19)	0.58850 (13)	0.0599 (8)	
C9'	0.3769 (2)	0.5086 (2)	0.66671 (15)	0.0788 (11)	
C10'	0.3577 (3)	0.4526 (3)	0.72053 (16)	0.0867 (11)	
C11'	0.3086 (2)	0.3547 (3)	0.70069 (14)	0.0799 (11)	
C12'	0.2770 (2)	0.3136 (2)	0.62795 (12)	0.0648 (9)	
C13'	0.29286 (18)	0.37098 (17)	0.56685 (12)	0.0512 (8)	
C14'	0.26145 (17)	0.33231 (16)	0.48937 (11)	0.0471 (7)	
C15'	0.20202 (17)	0.22763 (17)	0.47349 (11)	0.0487 (7)	
C16'	0.27389 (18)	0.12751 (16)	0.47554 (11)	0.0494 (7)	
C17'	0.40112 (18)	0.14129 (16)	0.48928 (12)	0.0494 (7)	
C18'	0.4458 (2)	0.16801 (19)	0.43213 (13)	0.0627 (9)	
C19'	0.5634 (2)	0.1823 (2)	0.44889 (17)	0.0704 (10)	
C20'	0.6365 (2)	0.1699 (2)	0.52095 (17)	0.0746 (10)	
C21'	0.5956 (2)	0.1404 (2)	0.57790 (15)	0.0713 (10)	
C22'	0.47776 (19)	0.12735 (18)	0.56074 (13)	0.0560 (8)	
C23'	0.3361 (2)	0.04727 (18)	0.60154 (13)	0.0579 (8)	
C24'	0.3182 (2)	−0.0088 (2)	0.65867 (15)	0.0750 (10)	
C25'	0.2170 (3)	−0.0663 (2)	0.64169 (19)	0.0885 (12)	
C26'	0.1382 (3)	−0.0691 (2)	0.56960 (19)	0.0885 (12)	
C27'	0.1551 (2)	−0.0086 (2)	0.51410 (16)	0.0751 (10)	
C28'	0.25504 (19)	0.05271 (17)	0.53059 (13)	0.0550 (8)	
C29'	0.19331 (19)	0.10879 (18)	0.39218 (13)	0.0541 (8)	
C30'	0.03498 (18)	0.23067 (17)	0.34005 (12)	0.0511 (7)	
C31'	−0.0381 (2)	0.29768 (18)	0.36066 (13)	0.0569 (8)	
C32'	−0.1302 (2)	0.3351 (2)	0.30565 (14)	0.0655 (9)	
C33'	−0.1550 (2)	0.30523 (19)	0.22879 (14)	0.0617 (8)	
C34'	−0.0870 (2)	0.2385 (2)	0.20754 (13)	0.0656 (9)	
C35'	0.0076 (2)	0.2021 (2)	0.26307 (13)	0.0627 (9)	
C36'	−0.0904 (3)	0.3752 (4)	0.46276 (19)	0.1243 (18)	
C37'	−0.2853 (2)	0.3143 (3)	0.10074 (15)	0.0922 (13)	
H2	0.60300	0.68540	−0.07150	0.0860*	
H3	0.45330	0.70620	−0.17790	0.1050*	
H4	0.46580	0.82340	−0.26730	0.1240*	
H5	0.63500	0.90930	−0.24970	0.1150*	
H7	0.83800	0.94470	−0.17720	0.0940*	
H9	1.04160	0.97970	−0.11160	0.1010*	
H10	1.20170	0.95660	−0.01390	0.1100*	
H11	1.19890	0.84980	0.08400	0.1050*	
H12	1.03040	0.76800	0.07980	0.0850*	
H15	0.90780	0.66930	0.05290	0.0600*	
H18	0.55550	0.75980	0.03960	0.0960*	
H19	0.46910	0.91120	0.04690	0.1220*	
H20	0.57780	1.05220	0.11550	0.1350*	
H21	0.77280	1.03890	0.17880	0.1220*	
H24	1.06930	0.85430	0.30280	0.1210*	
H25	1.13500	0.70090	0.35200	0.1300*	
H26	1.03550	0.55110	0.29780	0.1060*	
H27	0.87580	0.55240	0.18950	0.0810*	
H32	0.85240	0.40420	−0.15440	0.0780*	
H34	0.54840	0.33500	−0.12940	0.0830*	
H35	0.57730	0.46940	−0.04150	0.0760*	
H36A	1.02030	0.50300	−0.09940	0.1340*	
H36B	1.03700	0.62180	−0.08470	0.1340*	
H36C	0.93600	0.57480	−0.15540	0.1340*	
H37A	0.58720	0.18070	−0.17150	0.1800*	
H37B	0.60740	0.14680	−0.24820	0.1800*	
H37C	0.52950	0.23880	−0.24600	0.1800*	
H2'	0.23610	0.29250	0.33860	0.0760*	
H3'	0.28070	0.39540	0.25600	0.0920*	
H4'	0.35730	0.55940	0.29450	0.1050*	
H5'	0.39370	0.61980	0.41710	0.0930*	
H7'	0.39640	0.59440	0.54610	0.0830*	
H9'	0.41140	0.57350	0.68040	0.0950*	
H10'	0.37710	0.47900	0.77080	0.1040*	
H11'	0.29700	0.31590	0.73870	0.0960*	
H12'	0.24470	0.24770	0.61710	0.0780*	
H15'	0.15560	0.22010	0.50710	0.0580*	
H18'	0.39630	0.17630	0.38260	0.0750*	
H19'	0.59250	0.20040	0.41060	0.0840*	
H20'	0.71520	0.18150	0.53180	0.0900*	
H21'	0.64580	0.12950	0.62670	0.0860*	
H24'	0.37310	−0.00760	0.70730	0.0900*	
H25'	0.20240	−0.10320	0.67950	0.1060*	
H26'	0.07260	−0.11180	0.55760	0.1060*	
H27'	0.09940	−0.00910	0.46570	0.0900*	
H32'	−0.17680	0.38100	0.31990	0.0790*	
H34'	−0.10370	0.21740	0.15620	0.0790*	
H35'	0.05430	0.15690	0.24810	0.0750*	
H36D	−0.09370	0.44320	0.44510	0.1870*	
H36E	−0.06660	0.37820	0.51780	0.1870*	
H36F	−0.16510	0.34170	0.44210	0.1870*	
H37D	−0.22620	0.33160	0.07960	0.1380*	
H37E	−0.35470	0.34700	0.07340	0.1380*	
H37F	−0.29930	0.24170	0.09610	0.1380*	

### Atomic displacement parameters (Å^2^)

**Table:**

	*U*^11^	*U*^22^	*U*^33^	*U*^12^	*U*^13^	*U*^23^
O1	0.1113 (18)	0.0758 (14)	0.0818 (13)	−0.0323 (12)	0.0361 (13)	−0.0047 (11)
O2	0.0648 (10)	0.0836 (12)	0.0618 (9)	−0.0163 (9)	0.0331 (9)	0.0036 (8)
O3	0.0656 (11)	0.0824 (12)	0.0809 (11)	−0.0140 (9)	0.0425 (10)	−0.0095 (9)
O4	0.1228 (17)	0.0850 (14)	0.0636 (11)	−0.0187 (12)	0.0334 (12)	−0.0161 (10)
N1	0.0546 (11)	0.0603 (12)	0.0465 (10)	−0.0093 (9)	0.0238 (9)	−0.0006 (9)
C1	0.0729 (17)	0.0651 (15)	0.0457 (12)	0.0114 (12)	0.0269 (13)	0.0056 (11)
C2	0.0638 (17)	0.097 (2)	0.0543 (14)	0.0118 (14)	0.0179 (14)	0.0041 (14)
C3	0.080 (2)	0.112 (3)	0.0657 (17)	0.0220 (17)	0.0150 (16)	0.0024 (17)
C4	0.107 (3)	0.129 (3)	0.0591 (18)	0.040 (2)	0.0062 (18)	0.0093 (19)
C5	0.143 (3)	0.093 (2)	0.0521 (16)	0.031 (2)	0.029 (2)	0.0157 (15)
C6	0.105 (2)	0.0663 (16)	0.0495 (14)	0.0217 (15)	0.0348 (15)	0.0095 (12)
C7	0.133 (3)	0.0616 (16)	0.0611 (16)	0.0122 (16)	0.0572 (19)	0.0134 (13)
C8	0.105 (2)	0.0530 (14)	0.0681 (16)	0.0006 (14)	0.0545 (17)	0.0056 (12)
C9	0.120 (3)	0.0626 (17)	0.095 (2)	−0.0163 (18)	0.072 (2)	−0.0002 (16)
C10	0.108 (3)	0.0705 (19)	0.120 (3)	−0.0249 (19)	0.075 (2)	−0.0120 (19)
C11	0.080 (2)	0.080 (2)	0.110 (2)	−0.0186 (16)	0.0430 (18)	−0.0053 (17)
C12	0.0687 (17)	0.0655 (17)	0.0817 (17)	−0.0126 (13)	0.0309 (16)	0.0069 (14)
C13	0.0719 (16)	0.0488 (13)	0.0608 (14)	−0.0006 (11)	0.0365 (14)	0.0026 (11)
C14	0.0657 (15)	0.0553 (14)	0.0488 (12)	−0.0004 (11)	0.0291 (12)	0.0045 (10)
C15	0.0532 (13)	0.0542 (13)	0.0465 (11)	−0.0024 (10)	0.0212 (10)	0.0058 (10)
C16	0.0626 (14)	0.0605 (14)	0.0463 (12)	−0.0084 (11)	0.0267 (11)	0.0038 (10)
C17	0.0811 (19)	0.0679 (17)	0.0537 (13)	−0.0001 (14)	0.0385 (14)	0.0096 (12)
C18	0.095 (2)	0.085 (2)	0.0729 (17)	0.0188 (16)	0.0444 (17)	0.0165 (15)
C19	0.120 (3)	0.109 (3)	0.096 (2)	0.038 (2)	0.058 (2)	0.032 (2)
C20	0.189 (4)	0.078 (2)	0.113 (3)	0.037 (3)	0.103 (3)	0.022 (2)
C21	0.164 (4)	0.072 (2)	0.098 (2)	0.001 (2)	0.082 (3)	0.0077 (18)
C22	0.108 (2)	0.0608 (18)	0.0696 (17)	−0.0034 (17)	0.0513 (18)	0.0068 (14)
C23	0.083 (2)	0.086 (2)	0.0575 (15)	−0.0246 (16)	0.0288 (15)	−0.0010 (14)
C24	0.098 (3)	0.124 (3)	0.0698 (19)	−0.042 (2)	0.0188 (19)	−0.016 (2)
C25	0.093 (3)	0.153 (4)	0.0645 (19)	−0.016 (3)	0.0070 (18)	0.003 (2)
C26	0.084 (2)	0.117 (3)	0.0599 (16)	0.0094 (19)	0.0168 (16)	0.0128 (17)
C27	0.0695 (17)	0.0849 (19)	0.0496 (13)	−0.0026 (14)	0.0209 (13)	0.0054 (13)
C28	0.0607 (14)	0.0778 (17)	0.0451 (12)	−0.0137 (13)	0.0241 (12)	0.0013 (12)
C29	0.0546 (13)	0.0630 (14)	0.0466 (12)	0.0001 (11)	0.0223 (11)	0.0086 (10)
C30	0.0540 (13)	0.0620 (14)	0.0415 (11)	−0.0062 (11)	0.0167 (11)	0.0043 (10)
C31	0.0575 (14)	0.0598 (14)	0.0479 (12)	−0.0020 (11)	0.0184 (11)	0.0077 (11)
C32	0.0744 (17)	0.0748 (17)	0.0505 (13)	−0.0006 (14)	0.0278 (13)	0.0047 (12)
C33	0.0894 (19)	0.0705 (17)	0.0401 (12)	−0.0072 (14)	0.0166 (13)	−0.0009 (12)
C34	0.0692 (16)	0.0825 (19)	0.0500 (13)	−0.0185 (14)	0.0153 (13)	−0.0036 (13)
C35	0.0582 (15)	0.0841 (18)	0.0488 (13)	−0.0129 (13)	0.0194 (12)	0.0000 (12)
C36	0.0777 (19)	0.119 (3)	0.0907 (19)	−0.0113 (17)	0.0545 (17)	0.0046 (18)
C37	0.129 (3)	0.109 (3)	0.099 (2)	−0.031 (2)	0.016 (2)	−0.047 (2)
O1'	0.0662 (11)	0.0938 (13)	0.0491 (9)	−0.0157 (9)	0.0113 (8)	0.0045 (9)
O2'	0.0773 (12)	0.0655 (11)	0.0663 (10)	0.0034 (9)	0.0125 (9)	−0.0114 (9)
O3'	0.0658 (11)	0.1052 (15)	0.0563 (10)	0.0190 (10)	0.0164 (9)	−0.0092 (9)
O4'	0.0755 (13)	0.0963 (15)	0.0741 (12)	0.0140 (11)	−0.0030 (10)	0.0089 (11)
N1'	0.0498 (11)	0.0558 (12)	0.0479 (10)	0.0009 (9)	0.0129 (9)	0.0013 (8)
C1'	0.0481 (12)	0.0543 (13)	0.0516 (12)	0.0068 (10)	0.0174 (10)	0.0113 (10)
C2'	0.0618 (15)	0.0791 (18)	0.0511 (13)	−0.0042 (13)	0.0204 (12)	0.0110 (12)
C3'	0.0785 (18)	0.095 (2)	0.0589 (15)	0.0021 (16)	0.0243 (14)	0.0257 (14)
C4'	0.081 (2)	0.102 (2)	0.083 (2)	−0.0029 (17)	0.0292 (17)	0.0449 (18)
C5'	0.0785 (19)	0.0649 (17)	0.092 (2)	−0.0020 (14)	0.0286 (16)	0.0282 (15)
C6'	0.0566 (14)	0.0554 (14)	0.0704 (15)	0.0062 (11)	0.0226 (12)	0.0142 (12)
C7'	0.0702 (17)	0.0518 (15)	0.0819 (18)	−0.0039 (12)	0.0226 (14)	−0.0032 (13)
C8'	0.0584 (15)	0.0594 (15)	0.0612 (14)	0.0089 (11)	0.0198 (12)	−0.0069 (12)
C9'	0.0797 (19)	0.0786 (19)	0.0730 (17)	0.0034 (15)	0.0218 (15)	−0.0240 (15)
C10'	0.099 (2)	0.105 (2)	0.0563 (15)	0.0159 (18)	0.0278 (16)	−0.0171 (16)
C11'	0.094 (2)	0.101 (2)	0.0542 (15)	0.0133 (17)	0.0372 (15)	0.0015 (15)
C12'	0.0734 (17)	0.0765 (17)	0.0502 (13)	0.0039 (13)	0.0281 (13)	0.0024 (12)
C13'	0.0494 (13)	0.0556 (14)	0.0516 (12)	0.0088 (10)	0.0205 (11)	0.0004 (10)
C14'	0.0472 (12)	0.0501 (12)	0.0467 (11)	0.0056 (9)	0.0187 (10)	0.0041 (9)
C15'	0.0473 (12)	0.0575 (13)	0.0411 (11)	−0.0020 (10)	0.0144 (10)	0.0040 (10)
C16'	0.0496 (13)	0.0483 (12)	0.0487 (12)	0.0013 (10)	0.0138 (10)	0.0061 (10)
C17'	0.0506 (13)	0.0443 (12)	0.0530 (13)	0.0010 (10)	0.0171 (11)	0.0004 (10)
C18'	0.0663 (16)	0.0672 (16)	0.0590 (14)	−0.0010 (12)	0.0270 (13)	0.0026 (12)
C19'	0.0698 (17)	0.0685 (17)	0.0844 (19)	−0.0041 (13)	0.0427 (16)	−0.0060 (14)
C20'	0.0550 (16)	0.0741 (18)	0.097 (2)	−0.0088 (13)	0.0308 (16)	−0.0112 (15)
C21'	0.0563 (16)	0.0792 (19)	0.0709 (16)	−0.0036 (13)	0.0122 (13)	−0.0038 (14)
C22'	0.0526 (14)	0.0565 (14)	0.0561 (13)	−0.0041 (11)	0.0151 (12)	0.0003 (11)
C23'	0.0559 (14)	0.0592 (14)	0.0581 (14)	−0.0017 (11)	0.0176 (12)	0.0101 (11)
C24'	0.0860 (19)	0.0779 (18)	0.0648 (15)	0.0094 (15)	0.0270 (15)	0.0241 (13)
C25'	0.091 (2)	0.090 (2)	0.096 (2)	0.0003 (17)	0.043 (2)	0.0369 (18)
C26'	0.074 (2)	0.086 (2)	0.111 (2)	−0.0126 (16)	0.0361 (19)	0.0298 (18)
C27'	0.0599 (16)	0.0746 (18)	0.0873 (18)	−0.0075 (13)	0.0183 (14)	0.0225 (15)
C28'	0.0501 (13)	0.0529 (13)	0.0622 (14)	0.0029 (10)	0.0179 (12)	0.0110 (11)
C29'	0.0556 (14)	0.0513 (14)	0.0535 (13)	−0.0021 (11)	0.0160 (11)	0.0006 (11)
C30'	0.0459 (12)	0.0553 (13)	0.0505 (12)	−0.0011 (10)	0.0133 (11)	0.0075 (10)
C31'	0.0546 (14)	0.0588 (14)	0.0554 (14)	−0.0020 (11)	0.0160 (12)	0.0021 (11)
C32'	0.0570 (15)	0.0667 (16)	0.0686 (16)	0.0081 (12)	0.0150 (13)	0.0013 (13)
C33'	0.0518 (14)	0.0608 (15)	0.0635 (15)	−0.0007 (12)	0.0063 (12)	0.0105 (12)
C34'	0.0647 (16)	0.0765 (18)	0.0497 (13)	−0.0015 (14)	0.0109 (13)	0.0048 (12)
C35'	0.0631 (15)	0.0724 (17)	0.0511 (13)	0.0076 (12)	0.0167 (12)	0.0010 (12)
C36'	0.106 (3)	0.180 (4)	0.086 (2)	0.056 (2)	0.031 (2)	−0.025 (2)
C37'	0.0740 (19)	0.125 (3)	0.0639 (17)	−0.0091 (18)	0.0028 (15)	0.0243 (17)

### Geometric parameters (Å, °)

**Table:**

O1—C22	1.378 (4)	C27—H27	0.9300
O1—C23	1.378 (3)	C32—H32	0.9300
O2—C29	1.217 (3)	C34—H34	0.9300
O3—C31	1.369 (3)	C35—H35	0.9300
O3—C36	1.423 (3)	C36—H36C	0.9600
O4—C33	1.363 (3)	C36—H36B	0.9600
O4—C37	1.423 (5)	C36—H36A	0.9600
O1'—C23'	1.384 (3)	C37—H37A	0.9600
O1'—C22'	1.393 (3)	C37—H37B	0.9600
O2'—C29'	1.220 (3)	C37—H37C	0.9600
O3'—C31'	1.374 (3)	C1'—C2'	1.439 (3)
O3'—C36'	1.419 (5)	C1'—C6'	1.435 (3)
O4'—C33'	1.368 (3)	C1'—C14'	1.405 (3)
O4'—C37'	1.419 (3)	C2'—C3'	1.361 (4)
N1—C15	1.485 (3)	C3'—C4'	1.397 (5)
N1—C29	1.364 (3)	C4'—C5'	1.340 (4)
N1—C30	1.405 (3)	C5'—C6'	1.435 (4)
N1'—C15'	1.485 (3)	C6'—C7'	1.386 (4)
N1'—C29'	1.354 (3)	C7'—C8'	1.389 (4)
N1'—C30'	1.399 (3)	C8'—C9'	1.432 (3)
C1—C2	1.440 (3)	C8'—C13'	1.423 (3)
C1—C6	1.447 (3)	C9'—C10'	1.340 (4)
C1—C14	1.396 (3)	C10'—C11'	1.389 (5)
C2—C3	1.341 (4)	C11'—C12'	1.354 (3)
C3—C4	1.413 (5)	C12'—C13'	1.446 (3)
C4—C5	1.344 (6)	C13'—C14'	1.422 (3)
C5—C6	1.414 (5)	C14'—C15'	1.512 (3)
C6—C7	1.393 (5)	C15'—C16'	1.597 (3)
C7—C8	1.376 (4)	C16'—C17'	1.508 (3)
C8—C9	1.431 (5)	C16'—C28'	1.511 (3)
C8—C13	1.434 (4)	C16'—C29'	1.539 (3)
C9—C10	1.315 (5)	C17'—C18'	1.395 (3)
C10—C11	1.417 (4)	C17'—C22'	1.370 (3)
C11—C12	1.372 (4)	C18'—C19'	1.384 (4)
C12—C13	1.406 (4)	C19'—C20'	1.359 (4)
C13—C14	1.434 (3)	C20'—C21'	1.374 (4)
C14—C15	1.515 (3)	C21'—C22'	1.384 (4)
C15—C16	1.610 (3)	C23'—C24'	1.385 (4)
C16—C17	1.486 (4)	C23'—C28'	1.368 (3)
C16—C28	1.511 (3)	C24'—C25'	1.378 (4)
C16—C29	1.528 (3)	C25'—C26'	1.364 (5)
C17—C22	1.394 (4)	C26'—C27'	1.389 (4)
C17—C18	1.395 (4)	C27'—C28'	1.393 (4)
C18—C19	1.373 (5)	C30'—C31'	1.402 (3)
C19—C20	1.392 (6)	C30'—C35'	1.383 (3)
C20—C21	1.380 (7)	C31'—C32'	1.368 (3)
C21—C22	1.360 (5)	C32'—C33'	1.388 (3)
C23—C28	1.387 (4)	C33'—C34'	1.359 (4)
C23—C24	1.394 (4)	C34'—C35'	1.385 (3)
C24—C25	1.368 (6)	C2'—H2'	0.9300
C25—C26	1.373 (6)	C3'—H3'	0.9300
C26—C27	1.378 (4)	C4'—H4'	0.9300
C27—C28	1.390 (4)	C5'—H5'	0.9300
C30—C31	1.409 (3)	C7'—H7'	0.9300
C30—C35	1.379 (3)	C9'—H9'	0.9300
C31—C32	1.361 (3)	C10'—H10'	0.9300
C32—C33	1.391 (4)	C11'—H11'	0.9300
C33—C34	1.384 (4)	C12'—H12'	0.9300
C34—C35	1.366 (3)	C15'—H15'	0.9800
C2—H2	0.9300	C18'—H18'	0.9300
C3—H3	0.9300	C19'—H19'	0.9300
C4—H4	0.9300	C20'—H20'	0.9300
C5—H5	0.9300	C21'—H21'	0.9300
C7—H7	0.9300	C24'—H24'	0.9300
C9—H9	0.9300	C25'—H25'	0.9300
C10—H10	0.9300	C26'—H26'	0.9300
C11—H11	0.9300	C27'—H27'	0.9300
C12—H12	0.9300	C32'—H32'	0.9300
C15—H15	0.9800	C34'—H34'	0.9300
C18—H18	0.9300	C35'—H35'	0.9300
C19—H19	0.9300	C36'—H36D	0.9600
C20—H20	0.9300	C36'—H36E	0.9600
C21—H21	0.9300	C36'—H36F	0.9600
C24—H24	0.9300	C37'—H37D	0.9600
C25—H25	0.9300	C37'—H37E	0.9600
C26—H26	0.9300	C37'—H37F	0.9600
			
O1'···C37^i^	3.195 (4)	C30···H2	2.7100
O2···C18	3.354 (3)	C30'···H2'	2.6000
O2···C35	3.132 (3)	C30'···H26'^viii^	3.0900
O2···C34^ii^	3.409 (3)	C31'···H26'^viii^	3.0200
O2···C35^ii^	3.212 (3)	C32···H36C	2.8000
O2'···C35'	3.106 (3)	C32···H36A	2.8100
O2'···C27'	3.416 (3)	C32···H4'^ii^	3.0400
O3···N1	2.705 (3)	C32'···H36F	2.6900
O3···C13	3.366 (3)	C32'···H36D	2.7800
O3···O3^iii^	3.117 (2)	C33···H11^iii^	3.0700
O3···C36^iii^	3.377 (4)	C33···H4'^ii^	2.8700
O3···C15	2.718 (3)	C34···H37C	2.8300
O3···C14	2.975 (3)	C34···H37A	2.6800
O3'···C14'	3.161 (3)	C34'···H37F	2.7500
O3'···C15'	2.796 (3)	C34'···H21^vii^	3.0300
O3'···N1'	2.735 (3)	C34'···H37D	2.7700
O1···H9^iv^	2.5600	C35···H37E^vi^	2.9300
O1'···H37B^i^	2.6300	C35···H2	3.0500
O1'···H37C^i^	2.8900	C35'···H2'	2.9000
O2···H10'^v^	2.7100	C36···H32	2.5700
O2···H37E^vi^	2.8800	C36'···H32'	2.5100
O2···H18	2.8500	C37···H34	2.5300
O2···H27	2.9100	C37···H11^iii^	3.0100
O2···H35^ii^	2.5200	C37'···H34'	2.5300
O2···H35	2.5800	C37'···H3^ix^	2.8900
O2'···H27'	2.9200	H2···C18	3.0400
O2'···H24^vii^	2.7700	H2···N1	2.3300
O2'···H35'	2.5200	H2···C15	2.8000
O3···H36A^iii^	2.9100	H2···C29	2.7500
O3···H15	2.4100	H2···C30	2.7100
O3'···H26'^viii^	2.8300	H2···C35	3.0500
O3'···H15'	2.4600	H2···H18	2.4800
O4···H4'^ii^	2.8500	H2'···C35'	2.9000
O4···H11^iii^	2.7000	H2'···H18'	2.4600
O4'···H3^ix^	2.5900	H2'···C15'	2.8200
N1···C2	2.964 (3)	H2'···C29'	2.7500
N1···O3	2.705 (3)	H2'···N1'	2.3100
N1'···C2'	2.957 (3)	H2'···C30'	2.6000
N1'···O3'	2.735 (3)	H3···C37'^ix^	2.8900
N1···H2	2.3300	H3···O4'^ix^	2.5900
N1'···H2'	2.3100	H4···H24'^xv^	2.5100
C1···C30	3.597 (3)	H4···C24'^xv^	2.9700
C1'···C18'	3.565 (3)	H4···H19'^ii^	2.5100
C1'···C30'	3.591 (3)	H4'···O4^ii^	2.8500
C2···C30	3.261 (3)	H4'···C33^ii^	2.8700
C2···C29	3.544 (3)	H4'···C32^ii^	3.0400
C2···N1	2.964 (3)	H5···H18'^ii^	2.5600
C2···C18	3.425 (4)	H5···H7	2.4400
C2'···C18'	3.395 (4)	H5'···C20'^v^	3.0200
C2'···C29'	3.522 (4)	H5'···H7'	2.4100
C2'···C30'	3.201 (3)	H7···H9	2.4200
C2'···N1'	2.957 (3)	H7···H35'^ii^	2.5200
C4'···C9'^v^	3.481 (4)	H7···H5	2.4400
C5'···C7'^v^	3.403 (4)	H7'···C19'^v^	2.9500
C5'···C8'^v^	3.573 (4)	H7'···H5'	2.4100
C7···C11^iv^	3.522 (4)	H7'···H9'	2.4600
C7···C10^iv^	3.537 (4)	H9···H7	2.4200
C7'···C5'^v^	3.403 (4)	H9···O1^iv^	2.5600
C8···C10^iv^	3.274 (4)	H9···C22^iv^	3.0000
C8···C11^iv^	3.555 (4)	H9'···H7'	2.4600
C8···C9^iv^	3.514 (4)	H10···C21^iv^	3.0600
C8'···C5'^v^	3.573 (4)	H10'···O2^v^	2.7100
C9···C34'^ii^	3.506 (4)	H11···C33^iii^	3.0700
C9···C10^iv^	3.588 (5)	H11···C37^iii^	3.0100
C9···C13^iv^	3.550 (4)	H11···O4^iii^	2.7000
C9···C11^iv^	3.592 (5)	H11'···C28^v^	3.0200
C9···C9^iv^	3.541 (5)	H12···C15	2.5200
C9···C8^iv^	3.514 (4)	H12···H15	1.8900
C9···C12^iv^	3.550 (4)	H12'···C15'	2.5300
C9'···C4'^v^	3.481 (4)	H12'···H15'	1.9800
C10···C8^iv^	3.274 (4)	H12'···C23'	2.9300
C10···C7^iv^	3.537 (4)	H12'···C28'	2.9900
C10···C13^iv^	3.581 (4)	H15···O3	2.4100
C10···C9^iv^	3.588 (5)	H15···H12	1.8900
C11···C9^iv^	3.592 (5)	H15···C12	2.4200
C11···C7^iv^	3.522 (4)	H15···C27	3.0200
C11···C8^iv^	3.555 (4)	H15···H36A^iii^	2.5300
C12···C9^iv^	3.550 (4)	H15'···O3'	2.4600
C13···C10^iv^	3.581 (4)	H15'···C12'	2.4900
C13···C9^iv^	3.550 (4)	H15'···C27'	3.0100
C13···O3	3.366 (3)	H15'···H12'	1.9800
C14···O3	2.975 (3)	H18···O2	2.8500
C14···C31	3.599 (3)	H18···C2	3.0300
C14'···O3'	3.161 (3)	H18···C29	2.6600
C14'···C18'	3.542 (3)	H18···H2	2.4800
C15···O3	2.718 (3)	H18'···C29'	2.7000
C15'···O3'	2.796 (3)	H18'···H2'	2.4600
C18···O2	3.354 (3)	H18'···C2'	2.8600
C18···C2	3.425 (4)	H18'···H5^ii^	2.5600
C18'···C1'	3.565 (3)	H18'···C5^ii^	3.0500
C18'···C14'	3.542 (3)	H19'···H4^ii^	2.5100
C18'···C2'	3.395 (4)	H20'···C27'^x^	3.0500
C19'···C24'^x^	3.599 (4)	H21···C34'^xvi^	3.0300
C19'···C23'^x^	3.490 (4)	H21'···H37B^i^	2.5100
C20'···C27'^x^	3.568 (4)	H24···O2'^xvi^	2.7700
C20'···C28'^x^	3.467 (4)	H24'···H4^xiv^	2.5100
C23'···C19'^x^	3.490 (4)	H26'···C30'^viii^	3.0900
C24'···C19'^x^	3.599 (4)	H26'···O3'^viii^	2.8300
C27···C36^iii^	3.558 (4)	H26'···C31'^viii^	3.0200
C27'···C20'^x^	3.568 (4)	H27···H36A^iii^	2.5100
C27'···O2'	3.416 (3)	H27···O2	2.9100
C28'···C20'^x^	3.467 (4)	H27···C29	2.6300
C29···C2	3.544 (3)	H27'···C29'	2.5900
C29'···C2'	3.522 (4)	H27'···O2'	2.9200
C30···C2	3.261 (3)	H27'···C26'^viii^	3.0000
C30···C1	3.597 (3)	H32···C36	2.5700
C30'···C2'	3.201 (3)	H32···H36C	2.4500
C30'···C1'	3.591 (3)	H32···H36A	2.3200
C31···C14	3.599 (3)	H32'···H36D	2.3000
C34···O2^ii^	3.409 (3)	H32'···H36F	2.3000
C34'···C9^ii^	3.506 (4)	H32'···C10'^xiii^	3.0900
C35···O2^ii^	3.212 (3)	H32'···C9'^xiii^	2.9600
C35···C37'^vi^	3.500 (4)	H32'···C36'	2.5100
C35···O2	3.132 (3)	H34···C37	2.5300
C35'···O2'	3.106 (3)	H34···H37A	2.2600
C36···O3^iii^	3.377 (4)	H34···H37C	2.3900
C36···C27^iii^	3.558 (4)	H34'···C37'	2.5300
C37···O1'^xi^	3.195 (4)	H34'···H37F	2.3300
C37'···C35^xii^	3.500 (4)	H34'···C9^ii^	2.7700
C2···H18	3.0300	H34'···C10^ii^	3.0300
C2'···H18'	2.8600	H34'···H37D	2.3300
C5···H18'^ii^	3.0500	H35···O2	2.5800
C7···H35'^ii^	2.8500	H35···C29	2.8100
C9···H34'^ii^	2.7700	H35···O2^ii^	2.5200
C9'···H32'^xiii^	2.9600	H35'···C29'	2.7600
C10···H34'^ii^	3.0300	H35'···C7^ii^	2.8500
C10'···H32'^xiii^	3.0900	H35'···O2'	2.5200
C11'···H37C^i^	3.0400	H35'···H7^ii^	2.5200
C12···H15	2.4200	H36A···H32	2.3200
C12'···H15'	2.4900	H36A···O3^iii^	2.9100
C15···H2	2.8000	H36A···C32	2.8100
C15···H12	2.5200	H36A···C27^iii^	2.8500
C15'···H12'	2.5300	H36A···H15^iii^	2.5300
C15'···H2'	2.8200	H36A···H27^iii^	2.5100
C18···H2	3.0400	H36B···H37D^ii^	2.3700
C19···H37A^ii^	3.0900	H36C···C32	2.8000
C19'···H7'^v^	2.9500	H36C···H32	2.4500
C20'···H5'^v^	3.0200	H36D···C32'	2.7800
C21···H10^iv^	3.0600	H36D···H32'	2.3000
C22···H9^iv^	3.0000	H36F···C32'	2.6900
C23'···H12'	2.9300	H36F···H32'	2.3000
C24'···H4^xiv^	2.9700	H37A···C34	2.6800
C26'···H27'^viii^	3.0000	H37A···H34	2.2600
C27···H36A^iii^	2.8500	H37A···C19^ii^	3.0900
C27···H15	3.0200	H37B···O1'^xi^	2.6300
C27'···H20'^x^	3.0500	H37B···H21'^xi^	2.5100
C27'···H15'	3.0100	H37C···C34	2.8300
C28···H11'^v^	3.0200	H37C···H34	2.3900
C28'···H12'	2.9900	H37C···C11'^xi^	3.0400
C29···H35	2.8100	H37C···O1'^xi^	2.8900
C29···H18	2.6600	H37D···C34'	2.7700
C29···H27	2.6300	H37D···H34'	2.3300
C29···H2	2.7500	H37D···H36B^ii^	2.3700
C29'···H27'	2.5900	H37E···O2^xii^	2.8800
C29'···H18'	2.7000	H37E···C35^xii^	2.9300
C29'···H35'	2.7600	H37F···C34'	2.7500
C29'···H2'	2.7500	H37F···H34'	2.3300
			
C22—O1—C23	116.5 (2)	H37B—C37—H37C	110.00
C31—O3—C36	118.9 (2)	O4—C37—H37A	109.00
C33—O4—C37	117.5 (2)	O4—C37—H37B	109.00
C22'—O1'—C23'	116.38 (17)	O4—C37—H37C	109.00
C31'—O3'—C36'	117.2 (2)	H37A—C37—H37B	109.00
C33'—O4'—C37'	118.3 (2)	H37A—C37—H37C	109.00
C15—N1—C30	132.54 (18)	C2'—C1'—C6'	115.6 (2)
C15—N1—C29	95.21 (16)	C2'—C1'—C14'	125.4 (2)
C29—N1—C30	132.2 (2)	C6'—C1'—C14'	119.02 (19)
C15'—N1'—C29'	95.05 (17)	C1'—C2'—C3'	121.7 (2)
C15'—N1'—C30'	132.80 (18)	C2'—C3'—C4'	121.4 (2)
C29'—N1'—C30'	132.15 (19)	C3'—C4'—C5'	120.4 (3)
C2—C1—C14	125.5 (2)	C4'—C5'—C6'	120.6 (3)
C2—C1—C6	114.6 (2)	C1'—C6'—C5'	120.4 (2)
C6—C1—C14	120.0 (2)	C1'—C6'—C7'	119.9 (2)
C1—C2—C3	123.3 (3)	C5'—C6'—C7'	119.7 (2)
C2—C3—C4	120.8 (3)	C6'—C7'—C8'	122.1 (2)
C3—C4—C5	119.0 (3)	C7'—C8'—C9'	121.4 (2)
C4—C5—C6	122.3 (3)	C7'—C8'—C13'	118.7 (2)
C5—C6—C7	121.7 (3)	C9'—C8'—C13'	120.0 (2)
C1—C6—C5	119.9 (3)	C8'—C9'—C10'	121.3 (3)
C1—C6—C7	118.4 (2)	C9'—C10'—C11'	119.6 (3)
C6—C7—C8	122.8 (3)	C10'—C11'—C12'	122.3 (3)
C9—C8—C13	119.2 (3)	C11'—C12'—C13'	120.9 (3)
C7—C8—C9	120.9 (3)	C8'—C13'—C12'	116.0 (2)
C7—C8—C13	119.9 (3)	C8'—C13'—C14'	120.4 (2)
C8—C9—C10	121.4 (3)	C12'—C13'—C14'	123.6 (2)
C9—C10—C11	120.8 (3)	C1'—C14'—C13'	119.83 (19)
C10—C11—C12	119.5 (3)	C1'—C14'—C15'	125.23 (18)
C11—C12—C13	122.1 (3)	C13'—C14'—C15'	114.95 (18)
C12—C13—C14	124.6 (2)	N1'—C15'—C14'	120.43 (17)
C8—C13—C14	118.5 (2)	N1'—C15'—C16'	86.90 (15)
C8—C13—C12	116.9 (2)	C14'—C15'—C16'	120.73 (18)
C1—C14—C13	120.5 (2)	C15'—C16'—C17'	117.84 (18)
C1—C14—C15	125.0 (2)	C15'—C16'—C28'	111.94 (18)
C13—C14—C15	114.34 (18)	C15'—C16'—C29'	83.84 (15)
N1—C15—C14	119.47 (17)	C17'—C16'—C28'	109.19 (18)
N1—C15—C16	86.38 (16)	C17'—C16'—C29'	117.87 (18)
C14—C15—C16	123.19 (18)	C28'—C16'—C29'	114.37 (19)
C15—C16—C28	110.67 (19)	C16'—C17'—C18'	123.00 (19)
C15—C16—C17	118.62 (18)	C16'—C17'—C22'	119.4 (2)
C15—C16—C29	84.27 (16)	C18'—C17'—C22'	117.6 (2)
C17—C16—C28	109.82 (19)	C17'—C18'—C19'	120.3 (2)
C17—C16—C29	116.8 (2)	C18'—C19'—C20'	120.4 (3)
C28—C16—C29	114.7 (2)	C19'—C20'—C21'	120.8 (3)
C16—C17—C22	118.0 (2)	C20'—C21'—C22'	118.3 (2)
C18—C17—C22	117.5 (3)	O1'—C22'—C17'	122.0 (2)
C16—C17—C18	124.4 (2)	O1'—C22'—C21'	115.3 (2)
C17—C18—C19	120.7 (3)	C17'—C22'—C21'	122.6 (2)
C18—C19—C20	120.1 (4)	O1'—C23'—C24'	115.3 (2)
C19—C20—C21	119.9 (4)	O1'—C23'—C28'	121.9 (2)
C20—C21—C22	119.4 (4)	C24'—C23'—C28'	122.8 (2)
O1—C22—C17	121.0 (3)	C23'—C24'—C25'	118.4 (3)
O1—C22—C21	116.6 (3)	C24'—C25'—C26'	120.2 (3)
C17—C22—C21	122.4 (3)	C25'—C26'—C27'	120.5 (3)
O1—C23—C28	121.2 (2)	C26'—C27'—C28'	120.3 (3)
O1—C23—C24	117.6 (3)	C16'—C28'—C23'	119.5 (2)
C24—C23—C28	121.3 (3)	C16'—C28'—C27'	122.9 (2)
C23—C24—C25	119.3 (3)	C23'—C28'—C27'	117.5 (2)
C24—C25—C26	120.4 (3)	O2'—C29'—N1'	131.9 (2)
C25—C26—C27	120.3 (3)	O2'—C29'—C16'	134.0 (2)
C26—C27—C28	120.9 (3)	N1'—C29'—C16'	94.11 (17)
C23—C28—C27	117.7 (2)	N1'—C30'—C31'	122.48 (19)
C16—C28—C27	124.8 (2)	N1'—C30'—C35'	120.2 (2)
C16—C28—C23	117.4 (2)	C31'—C30'—C35'	117.3 (2)
N1—C29—C16	94.15 (18)	O3'—C31'—C30'	115.0 (2)
O2—C29—N1	131.3 (2)	O3'—C31'—C32'	124.5 (2)
O2—C29—C16	134.6 (2)	C30'—C31'—C32'	120.4 (2)
C31—C30—C35	118.1 (2)	C31'—C32'—C33'	120.8 (2)
N1—C30—C31	120.9 (2)	O4'—C33'—C32'	115.3 (2)
N1—C30—C35	121.0 (2)	O4'—C33'—C34'	124.8 (2)
O3—C31—C30	114.82 (19)	C32'—C33'—C34'	119.9 (2)
O3—C31—C32	124.8 (2)	C33'—C34'—C35'	119.4 (2)
C30—C31—C32	120.3 (2)	C30'—C35'—C34'	122.2 (2)
C31—C32—C33	120.4 (2)	C1'—C2'—H2'	119.00
C32—C33—C34	119.6 (2)	C3'—C2'—H2'	119.00
O4—C33—C34	125.1 (2)	C2'—C3'—H3'	119.00
O4—C33—C32	115.3 (2)	C4'—C3'—H3'	119.00
C33—C34—C35	119.6 (2)	C3'—C4'—H4'	120.00
C30—C35—C34	121.8 (2)	C5'—C4'—H4'	120.00
C3—C2—H2	118.00	C4'—C5'—H5'	120.00
C1—C2—H2	118.00	C6'—C5'—H5'	120.00
C2—C3—H3	120.00	C6'—C7'—H7'	119.00
C4—C3—H3	120.00	C8'—C7'—H7'	119.00
C3—C4—H4	120.00	C8'—C9'—H9'	119.00
C5—C4—H4	121.00	C10'—C9'—H9'	119.00
C6—C5—H5	119.00	C9'—C10'—H10'	120.00
C4—C5—H5	119.00	C11'—C10'—H10'	120.00
C6—C7—H7	119.00	C10'—C11'—H11'	119.00
C8—C7—H7	119.00	C12'—C11'—H11'	119.00
C8—C9—H9	119.00	C11'—C12'—H12'	120.00
C10—C9—H9	119.00	C13'—C12'—H12'	120.00
C11—C10—H10	120.00	N1'—C15'—H15'	109.00
C9—C10—H10	120.00	C14'—C15'—H15'	109.00
C10—C11—H11	120.00	C16'—C15'—H15'	109.00
C12—C11—H11	120.00	C17'—C18'—H18'	120.00
C11—C12—H12	119.00	C19'—C18'—H18'	120.00
C13—C12—H12	119.00	C18'—C19'—H19'	120.00
C14—C15—H15	109.00	C20'—C19'—H19'	120.00
N1—C15—H15	109.00	C19'—C20'—H20'	120.00
C16—C15—H15	109.00	C21'—C20'—H20'	120.00
C19—C18—H18	120.00	C20'—C21'—H21'	121.00
C17—C18—H18	120.00	C22'—C21'—H21'	121.00
C18—C19—H19	120.00	C23'—C24'—H24'	121.00
C20—C19—H19	120.00	C25'—C24'—H24'	121.00
C19—C20—H20	120.00	C24'—C25'—H25'	120.00
C21—C20—H20	120.00	C26'—C25'—H25'	120.00
C22—C21—H21	120.00	C25'—C26'—H26'	120.00
C20—C21—H21	120.00	C27'—C26'—H26'	120.00
C23—C24—H24	120.00	C26'—C27'—H27'	120.00
C25—C24—H24	120.00	C28'—C27'—H27'	120.00
C26—C25—H25	120.00	C31'—C32'—H32'	120.00
C24—C25—H25	120.00	C33'—C32'—H32'	120.00
C25—C26—H26	120.00	C33'—C34'—H34'	120.00
C27—C26—H26	120.00	C35'—C34'—H34'	120.00
C26—C27—H27	120.00	C30'—C35'—H35'	119.00
C28—C27—H27	120.00	C34'—C35'—H35'	119.00
C33—C32—H32	120.00	O3'—C36'—H36D	109.00
C31—C32—H32	120.00	O3'—C36'—H36E	109.00
C33—C34—H34	120.00	O3'—C36'—H36F	110.00
C35—C34—H34	120.00	H36D—C36'—H36E	109.00
C30—C35—H35	119.00	H36D—C36'—H36F	109.00
C34—C35—H35	119.00	H36E—C36'—H36F	109.00
H36A—C36—H36B	109.00	O4'—C37'—H37D	109.00
H36A—C36—H36C	109.00	O4'—C37'—H37E	109.00
O3—C36—H36A	109.00	O4'—C37'—H37F	109.00
O3—C36—H36B	109.00	H37D—C37'—H37E	109.00
O3—C36—H36C	110.00	H37D—C37'—H37F	110.00
H36B—C36—H36C	110.00	H37E—C37'—H37F	110.00
			
C23—O1—C22—C21	150.1 (3)	C24—C23—C28—C16	−171.4 (3)
C22—O1—C23—C24	−154.9 (3)	C28—C23—C24—C25	−2.4 (5)
C22—O1—C23—C28	25.5 (4)	C23—C24—C25—C26	−1.2 (5)
C23—O1—C22—C17	−29.0 (4)	C24—C25—C26—C27	2.4 (5)
C36—O3—C31—C32	15.5 (4)	C25—C26—C27—C28	−0.1 (5)
C36—O3—C31—C30	−164.2 (2)	C26—C27—C28—C16	172.3 (3)
C37—O4—C33—C32	179.7 (2)	C26—C27—C28—C23	−3.3 (4)
C37—O4—C33—C34	−2.3 (4)	C35—C30—C31—C32	−1.5 (3)
C22'—O1'—C23'—C28'	−20.5 (3)	N1—C30—C31—O3	−3.3 (3)
C23'—O1'—C22'—C21'	−155.5 (2)	N1—C30—C31—C32	177.1 (2)
C22'—O1'—C23'—C24'	159.9 (2)	C31—C30—C35—C34	1.5 (3)
C23'—O1'—C22'—C17'	24.3 (3)	C35—C30—C31—O3	178.1 (2)
C36'—O3'—C31'—C32'	8.0 (4)	N1—C30—C35—C34	−177.1 (2)
C36'—O3'—C31'—C30'	−171.1 (3)	O3—C31—C32—C33	179.2 (2)
C37'—O4'—C33'—C34'	5.4 (4)	C30—C31—C32—C33	−1.2 (4)
C37'—O4'—C33'—C32'	−174.9 (2)	C31—C32—C33—C34	4.1 (4)
C15—N1—C29—C16	−0.12 (18)	C31—C32—C33—O4	−177.8 (2)
C30—N1—C15—C16	−176.3 (2)	O4—C33—C34—C35	178.0 (2)
C29—N1—C15—C16	0.11 (17)	C32—C33—C34—C35	−4.1 (4)
C30—N1—C29—C16	176.4 (2)	C33—C34—C35—C30	1.4 (4)
C15—N1—C30—C35	−161.6 (2)	C6'—C1'—C14'—C15'	176.2 (2)
C29—N1—C30—C35	23.2 (4)	C14'—C1'—C6'—C7'	2.5 (4)
C29—N1—C30—C31	−155.4 (2)	C2'—C1'—C14'—C13'	175.1 (2)
C29—N1—C15—C14	−126.4 (2)	C14'—C1'—C6'—C5'	−179.0 (2)
C30—N1—C29—O2	−3.5 (4)	C6'—C1'—C14'—C13'	−4.1 (3)
C30—N1—C15—C14	57.2 (3)	C14'—C1'—C2'—C3'	179.7 (2)
C15—N1—C29—O2	−179.9 (3)	C2'—C1'—C6'—C5'	1.7 (3)
C15—N1—C30—C31	19.9 (3)	C2'—C1'—C6'—C7'	−176.8 (2)
C29'—N1'—C15'—C14'	121.7 (2)	C6'—C1'—C2'—C3'	−1.1 (4)
C29'—N1'—C30'—C35'	−21.0 (4)	C2'—C1'—C14'—C15'	−4.6 (4)
C29'—N1'—C30'—C31'	158.4 (2)	C1'—C2'—C3'—C4'	−0.2 (4)
C29'—N1'—C15'—C16'	−2.44 (17)	C2'—C3'—C4'—C5'	0.8 (4)
C30'—N1'—C15'—C16'	177.1 (2)	C3'—C4'—C5'—C6'	−0.1 (4)
C15'—N1'—C30'—C35'	159.7 (2)	C4'—C5'—C6'—C1'	−1.2 (4)
C30'—N1'—C29'—C16'	−177.0 (2)	C4'—C5'—C6'—C7'	177.4 (3)
C30'—N1'—C29'—O2'	2.2 (5)	C5'—C6'—C7'—C8'	−178.0 (2)
C30'—N1'—C15'—C14'	−58.8 (3)	C1'—C6'—C7'—C8'	0.5 (4)
C15'—N1'—C29'—O2'	−178.3 (3)	C6'—C7'—C8'—C9'	178.0 (2)
C15'—N1'—C30'—C31'	−20.9 (4)	C6'—C7'—C8'—C13'	−1.8 (4)
C15'—N1'—C29'—C16'	2.54 (18)	C7'—C8'—C13'—C14'	0.2 (4)
C2—C1—C6—C7	−177.5 (2)	C9'—C8'—C13'—C12'	−0.9 (3)
C6—C1—C14—C15	−176.3 (2)	C7'—C8'—C9'—C10'	179.6 (3)
C14—C1—C6—C7	1.5 (4)	C7'—C8'—C13'—C12'	179.0 (2)
C2—C1—C6—C5	−0.3 (4)	C13'—C8'—C9'—C10'	−0.5 (4)
C14—C1—C2—C3	−177.0 (3)	C9'—C8'—C13'—C14'	−179.7 (2)
C2—C1—C14—C15	2.6 (4)	C8'—C9'—C10'—C11'	1.6 (5)
C6—C1—C14—C13	−1.1 (3)	C9'—C10'—C11'—C12'	−1.2 (5)
C6—C1—C2—C3	1.8 (4)	C10'—C11'—C12'—C13'	−0.3 (5)
C2—C1—C14—C13	177.7 (2)	C11'—C12'—C13'—C14'	−179.9 (2)
C14—C1—C6—C5	178.6 (3)	C11'—C12'—C13'—C8'	1.3 (4)
C1—C2—C3—C4	−2.7 (5)	C8'—C13'—C14'—C15'	−177.4 (2)
C2—C3—C4—C5	1.9 (6)	C12'—C13'—C14'—C15'	3.9 (3)
C3—C4—C5—C6	−0.4 (6)	C8'—C13'—C14'—C1'	2.8 (3)
C4—C5—C6—C7	176.7 (3)	C12'—C13'—C14'—C1'	−175.9 (2)
C4—C5—C6—C1	−0.3 (5)	C13'—C14'—C15'—N1'	160.0 (2)
C5—C6—C7—C8	−177.6 (3)	C1'—C14'—C15'—C16'	85.7 (3)
C1—C6—C7—C8	−0.5 (4)	C13'—C14'—C15'—C16'	−94.0 (2)
C6—C7—C8—C9	178.8 (3)	C1'—C14'—C15'—N1'	−20.3 (3)
C6—C7—C8—C13	−0.9 (4)	N1'—C15'—C16'—C17'	120.49 (18)
C9—C8—C13—C14	−178.4 (2)	C14'—C15'—C16'—C17'	−3.4 (3)
C13—C8—C9—C10	−0.3 (4)	N1'—C15'—C16'—C28'	−111.67 (18)
C7—C8—C9—C10	−180.0 (3)	C14'—C15'—C16'—C29'	−121.76 (19)
C7—C8—C13—C12	−178.6 (3)	N1'—C15'—C16'—C29'	2.15 (15)
C9—C8—C13—C12	1.8 (4)	C14'—C15'—C16'—C28'	124.4 (2)
C7—C8—C13—C14	1.3 (4)	C15'—C16'—C28'—C23'	−101.0 (2)
C8—C9—C10—C11	−0.7 (5)	C29'—C16'—C28'—C23'	165.8 (2)
C9—C10—C11—C12	0.2 (5)	C29'—C16'—C28'—C27'	−17.5 (3)
C10—C11—C12—C13	1.4 (4)	C29'—C16'—C17'—C18'	19.7 (3)
C11—C12—C13—C14	177.8 (3)	C29'—C16'—C17'—C22'	−160.4 (2)
C11—C12—C13—C8	−2.3 (4)	C15'—C16'—C28'—C27'	75.7 (3)
C8—C13—C14—C15	175.4 (2)	C17'—C16'—C28'—C23'	31.3 (3)
C12—C13—C14—C15	−4.8 (3)	C17'—C16'—C28'—C27'	−152.0 (2)
C12—C13—C14—C1	179.5 (2)	C15'—C16'—C17'—C18'	−78.5 (3)
C8—C13—C14—C1	−0.3 (3)	C28'—C16'—C17'—C22'	−27.7 (3)
C13—C14—C15—C16	104.5 (2)	C15'—C16'—C17'—C22'	101.5 (2)
C1—C14—C15—C16	−80.0 (3)	C15'—C16'—C29'—O2'	178.5 (3)
C13—C14—C15—N1	−149.0 (2)	C17'—C16'—C29'—O2'	60.2 (4)
C1—C14—C15—N1	26.4 (3)	C17'—C16'—C29'—N1'	−120.7 (2)
C14—C15—C16—C17	5.6 (3)	C15'—C16'—C29'—N1'	−2.36 (17)
N1—C15—C16—C28	114.2 (2)	C28'—C16'—C17'—C18'	152.4 (2)
N1—C15—C16—C17	−117.6 (2)	C28'—C16'—C29'—N1'	109.0 (2)
C14—C15—C16—C28	−122.6 (2)	C28'—C16'—C29'—O2'	−70.1 (4)
N1—C15—C16—C29	−0.10 (15)	C16'—C17'—C22'—C21'	−178.9 (2)
C14—C15—C16—C29	123.1 (2)	C18'—C17'—C22'—O1'	−178.6 (2)
C29—C16—C28—C27	14.5 (3)	C16'—C17'—C22'—O1'	1.4 (3)
C15—C16—C29—O2	179.9 (3)	C18'—C17'—C22'—C21'	1.1 (4)
C28—C16—C17—C22	32.9 (3)	C22'—C17'—C18'—C19'	−1.8 (3)
C17—C16—C28—C23	−36.0 (3)	C16'—C17'—C18'—C19'	178.2 (2)
C29—C16—C28—C23	−169.9 (2)	C17'—C18'—C19'—C20'	0.4 (4)
C17—C16—C28—C27	148.4 (2)	C18'—C19'—C20'—C21'	1.8 (4)
C28—C16—C29—N1	−110.0 (2)	C19'—C20'—C21'—C22'	−2.4 (4)
C29—C16—C17—C18	−12.1 (3)	C20'—C21'—C22'—O1'	−179.3 (2)
C15—C16—C17—C22	−95.7 (3)	C20'—C21'—C22'—C17'	0.9 (4)
C15—C16—C28—C27	−78.8 (3)	C24'—C23'—C28'—C16'	171.0 (2)
C15—C16—C17—C18	86.5 (3)	C24'—C23'—C28'—C27'	−5.9 (4)
C17—C16—C29—O2	−60.8 (3)	O1'—C23'—C28'—C16'	−8.6 (3)
C17—C16—C29—N1	119.4 (2)	O1'—C23'—C28'—C27'	174.5 (2)
C28—C16—C29—O2	69.8 (4)	C28'—C23'—C24'—C25'	3.9 (4)
C29—C16—C17—C22	165.8 (2)	O1'—C23'—C24'—C25'	−176.5 (2)
C15—C16—C29—N1	0.11 (16)	C23'—C24'—C25'—C26'	1.5 (4)
C15—C16—C28—C23	96.9 (3)	C24'—C25'—C26'—C27'	−4.6 (5)
C28—C16—C17—C18	−144.9 (3)	C25'—C26'—C27'—C28'	2.5 (4)
C16—C17—C18—C19	179.9 (3)	C26'—C27'—C28'—C23'	2.6 (4)
C18—C17—C22—O1	176.0 (3)	C26'—C27'—C28'—C16'	−174.1 (2)
C16—C17—C22—O1	−2.0 (4)	N1'—C30'—C31'—C32'	178.5 (2)
C22—C17—C18—C19	2.1 (4)	C35'—C30'—C31'—O3'	177.1 (2)
C16—C17—C22—C21	179.1 (3)	C35'—C30'—C31'—C32'	−2.1 (4)
C18—C17—C22—C21	−2.9 (4)	N1'—C30'—C35'—C34'	−179.7 (2)
C17—C18—C19—C20	−0.2 (5)	C31'—C30'—C35'—C34'	0.9 (4)
C18—C19—C20—C21	−1.0 (6)	N1'—C30'—C31'—O3'	−2.3 (3)
C19—C20—C21—C22	0.2 (6)	O3'—C31'—C32'—C33'	−177.2 (2)
C20—C21—C22—O1	−177.2 (3)	C30'—C31'—C32'—C33'	1.9 (4)
C20—C21—C22—C17	1.8 (5)	C31'—C32'—C33'—C34'	−0.4 (4)
C24—C23—C28—C27	4.5 (4)	C31'—C32'—C33'—O4'	179.9 (2)
O1—C23—C24—C25	178.1 (3)	O4'—C33'—C34'—C35'	178.8 (2)
O1—C23—C28—C16	8.1 (4)	C32'—C33'—C34'—C35'	−0.9 (4)
O1—C23—C28—C27	−175.9 (2)	C33'—C34'—C35'—C30'	0.6 (4)

Symmetry codes: (i) *x*, *y*, *z*+1; (ii) −*x*+1, −*y*+1, −*z*; (iii) −*x*+2, −*y*+1, −*z*; (iv) −*x*+2, −*y*+2, −*z*; (v) −*x*+1, −*y*+1, −*z*+1; (vi) *x*+1, *y*, *z*; (vii) *x*−1, *y*−1, *z*; (viii) −*x*, −*y*, −*z*+1; (ix) −*x*, −*y*+1, −*z*; (x) −*x*+1, −*y*, −*z*+1; (xi) *x*, *y*, *z*−1; (xii) *x*−1, *y*, *z*; (xiii) −*x*, −*y*+1, −*z*+1; (xiv) *x*, *y*−1, *z*+1; (xv) *x*, *y*+1, *z*−1; (xvi) *x*+1, *y*+1, *z*.

### Hydrogen-bond geometry (Å, °)

**Table:**

*D*—H···*A*	*D*—H	H···*A*	*D*···*A*	*D*—H···*A*
C2—H2···N1	0.93	2.33	2.964 (3)	125
C2'—H2'···N1'	0.93	2.31	2.957 (3)	126
C3—H3···O4'^ix^	0.93	2.59	3.484 (4)	163
C9—H9···O1^iv^	0.93	2.56	3.458 (4)	164
C35—H35···O2	0.93	2.58	3.132 (3)	119
C35—H35···O2^ii^	0.93	2.52	3.212 (3)	131
C35'—H35'···O2'	0.93	2.52	3.106 (3)	121
C2—H2···Cg1	0.93	2.59	3.188 (3)	122
C2'—H2'···Cg15	0.93	2.62	3.196 (3)	121
C25'—H25'···Cg22^viii^	0.93	2.98	3.791 (3)	147

Symmetry codes: (ix) −*x*, −*y*+1, −*z*; (iv) −*x*+2, −*y*+2, −*z*; (ii) −*x*+1, −*y*+1, −*z*;  (viii) −*x*, −*y*, −*z*+1.

### Table 2 Comparision of the dihedral angles between the planes of the rings for molecules IA and IB.

(The prime suffix shows the similar planes and angles for the  molecule IB).

**Table:**

Plane1	Plane2	Angle(1/2)	Angle(1'/2')
A	B	80.83 (16)	81.34 (15)
A	C	67.20 (17)	72.70 (15)
A	D	67.97 (13)	65.71 (13)
A	E	22.23 (14)	20.88 (15)
B	C	32.34 (16)	26.90 (13)
B	D	46.25 (12)	41.50 (10)
B	E	77.80 (13)	79.39 (13)
C	D	67.09 (13)	62.22 (9)
C	E	45.50 (14)	52.74 (12)
D	E	79.35 (9)	76.25 (10)

The planes of the  molecule IA are A(N1/C15/C16/C29), B(C23–C28), C(C17–C22),  D(C1–C14) and E(C30–C35) .

### Table 3 The dihedral angles between the similar ring planes in molecules IA and IB

**Table:**

Plane	Plane	Angle
A	A'	86.92 (17)
B	B'	46.81 (14)
C	C'	88.40 (14)
D	D'	54.64 (6)
E	E'	65.35 (12)

The planes of the molecule IB are A'(N1'/C15'/C16'/C29'), B'(C23'–C28'), C'(C17'–C22'),  D'(C1'–C14') and E'(C30'–C35').
